# Cropland expansion links climate extremes and diets in Nigeria

**DOI:** 10.1126/sciadv.ado5541

**Published:** 2025-01-10

**Authors:** Bhoktear Khan, Piyush Mehta, Dongyang Wei, Hanan Abou Ali, Oluseun Adeluyi, Tunrayo Alabi, Olawale Olayide, John Uponi, Kyle Frankel Davis

**Affiliations:** ^1^ Department of Geography and Spatial Sciences, University of Delaware, Newark, DE, USA.; ^2^ National Space Research and Development Agency (NASRDA), Abuja, Nigeria.; ^3^ Geospatial Laboratory, International Institute of Tropical Agriculture (IITA), Ibadan, Nigeria.; ^4^ Department of Sustainability Studies, University of Ibadan, Ibadan, Nigeria.; ^5^ Department of Plant and Soil Sciences, University of Delaware, Newark, DE, USA.

## Abstract

Climate change threatens smallholder agriculture and food security in the Global South. While cropland expansion is often used to counter adverse climate effects despite ecological trade-offs, the benefits for diets and nutrition remain unclear. This study quantitatively examines relationships between climate anomalies, forest loss from cropland expansion, and dietary outcomes in Nigeria, Africa’s most populous country. Combining high-resolution data on forest cover and climate variables within random forest and panel regression models, we find that 25 to 31% of annual forest loss is linked to climate variability. Using georeferenced household survey data, we then find that changes in forest cover have a significant positive association with changes in child diet diversity—a key proxy of nutritional adequacy—while cropland expansion does not, suggesting that such forest conversions may be an ineffective climate adaptation strategy for improving nutrition. Our findings highlight the potential of nutrition-sensitive climate adaptation to enhance yields, promote nutritious cropping choices, and protect remaining forests.

## Teaser

Climate-associated cropland expansion does not compensate for the dietary benefits of forests.

## INTRODUCTION

Climate change poses a rising challenge to agricultural systems across the planet (*1*, *2*). The growing frequency and intensity of climate anomalies is leading to reduced yields (*1*, *3*), greater production instability (*3*), and cropping pattern migrations in response to shifting agro-climatic envelopes (*4*, *5*)—with critical implications for farmers and rural livelihoods. Potentially most vulnerable to these climatic effects are smallholders who—despite limited access to inputs and improved technologies (*6*, *7*)—are using a diverse toolkit of strategies to adapt to changing climate conditions. In addition to using strategies such as crop diversification (*8*), soil and water management (*9*), and improved crop varieties, among others (*8*, *10*), smallholders may also use cropland expansion as an adaptive strategy to offset crop yield losses from adverse climate conditions (and to thereby maintain levels of production) (*11*–*15*). Recent work has identified an association between adverse rainfall anomalies and cropland expansion in developing countries, particularly in regions with limited access to buffering infrastructure (*16*).

This cropland expansion can often occur at the expense of forests, particularly in Sub-Saharan Africa, where it is the primary and dominant driver of forest loss (*17*–*19*). While the decision to expand croplands in response to increasing climate variability and extremes may be primarily an economic one for smallholders, little is known about whether cropland expansion is associated with benefits for the food security and nutrition of local communities. On one hand, increased cropland area allows for increased food production, particularly staple and cash crops, across many countries (*20*), and this expansion can be related to improved food security outcomes through increasing production, enhancing incomes, and lowering prices (*21*). On the other hand, forests provide a diversity of food resources such as fruits, nuts, roots, and game, and there is a well-established correlation indicating that access to forests is positively associated with favorable nutritional outcomes (*22*–*25*). For instance, recent studies in Malawi (*26*) and Tanzania (*27*) found that higher forest cover and lower levels of forest loss were associated with higher fruit and vegetable intake and vitamin A adequacy. Other recent work found a positive association between forest regrowth and fruit and vegetable intake in Nigeria (*28*). More than three-quarters of existing studies on the topic have found that forests have a positive impact on food and nutrition security—due to both the direct collection of forest foods and the beneficial indirect effects of forest-based ecosystem services on agriculture (e.g., water provision, soil fertility, income opportunities, and fuelwood access for cooking) (*29*). Thus, while the replacement of forests with croplands clearly produces short-term economic benefits at the cost of biodiversity and carbon sequestration, there is limited understanding of how these land-use changes are associated with nutritional effects for local communities—who rely on a balance of both staple foods from croplands as well as many other nutritionally important products from forests—and the extent to which climate change may be altering this balance.

To address this knowledge gap, here we examine the extent to which deforestation due to cropland expansion is associated with climate anomalies, and how nutritional outcomes in terms of child dietary diversity are correlated with forest loss and cropland expansion. With rapid deforestation occurring as a result of cropland expansion, as well as being Africa’s most populous country and largest food producer (*30*), Nigeria presents the ideal focus country for investigating such dynamics between climate change, forests, and diets. To do so, we focus on its 20 southern states, which account for nearly all the country’s remaining forests. First, we use high-resolution gridded data on annual forest cover (1998 to 2021) (*31*) to quantify rates of deforestation and to characterize spatially detailed patterns of forest loss. Combining this information with a suite of climatic variables, we use random forest and panel fixed-effects regression models to evaluate the extent to which annual forest loss due to cropland expansion is explained by climate variability and extremes. Last, we integrate data on dietary intake, forest loss, cropland expansion, and a suite of additional agroecological, geographic, socioeconomic, and demographic control variables known to influence diets into mixed-effects and panel fixed-effects regression models to assess whether cropland expansion (via forest loss) is associated with local improvements in child diet diversity—a widely used proxy of nutritional adequacy (*32*). Better understanding these linkages can inform more effective strategies for simultaneously enhancing farmer productivity, climate adaptation, improving the nutrition of rural communities, and protecting remaining forests and can provide insights applicable to many other contexts where smallholder farming systems prevail.

## RESULTS

**Fig. 1.**
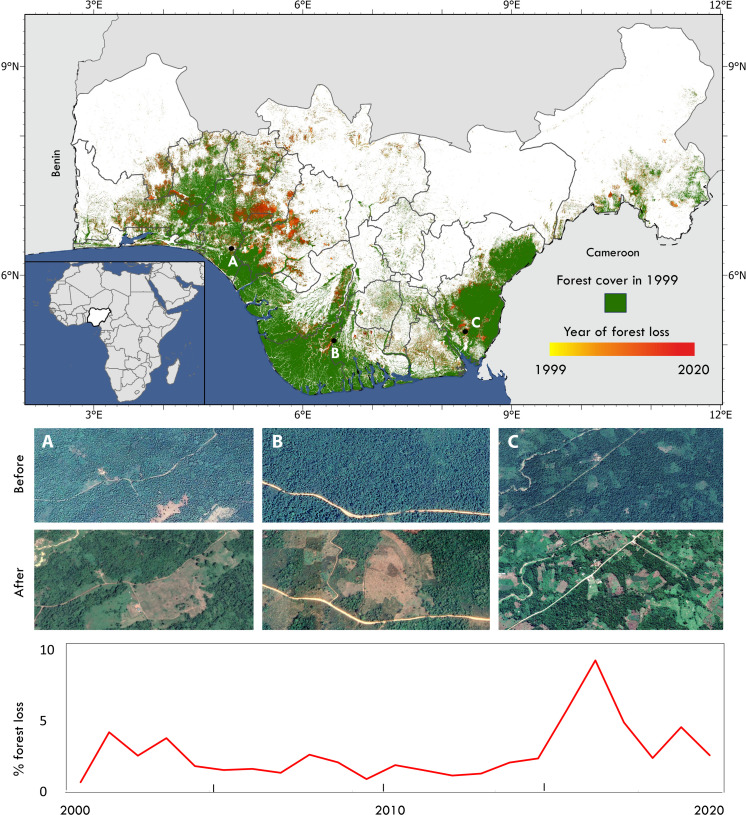
Trends of deforestation across southern Nigeria. Map shows the 20 southern states of Nigeria with forest cover and loss from 1999 to 2021. The accompanying line graph shows annual rates of forest loss over the 22-year period. Insets (**A** to **C**), sourced from Google Earth Pro, provide typical examples of land-use change from forest to cropland in three different parts of Nigeria: top and bottom: (A) 2005 and 2017, (B) 2007 and 2019, and (C) 2005 and 2015, respectively.

We estimate that 27% of southern Nigeria was covered by forests in 1998. Between 1998 and 2021, we found that 42% of these forests were lost, with enhanced rates of forest loss occurring in the most recent years (Fig. 1). While substantial forest loss has been widespread across the country’s remaining forests, hotspots of concentrated deforestation have occurred in Edo, Ondo, Osun, and Ogun (Fig. 1), and cross-classification confirms the replacement of forests with cropland (see e.g., Fig. 1, insets; Table 1).

**Table 1.** Percentage of deforested area converted to cropland (2000 to 2019). Cross-classification was performed for deforested areas immediately converted to cropland in the subsequent year and deforested areas converted to cropland within 5 years of forest loss [to account for more indirect pathways (e.g., forest to pasture to cropland)]. Given the well-known limitations of global cropland products in accurately classifying smallholder croplands (*66*, *67*) and the fact that each cropland product is trained using a different set of crops as groundtruth, we also assessed the fraction of forest-to-cropland conversion that occurred where both cropland products spatially agreed (i.e., “Agreement”) or where at least one of the products indicated the presence of cropland (i.e., “At least one”). The years selected in this table were based on the availability of cropland data; while ESA CCI cropland data are available annually over the study period, GLAD cropland data are only available for five time windows (2000 to 2003, 2004 to 2007, 2008 to 2011, 2012 to 2015, and 2016 to 2019).

Forest loss time period	% Deforested area immediately converted to cropland		% Deforested area converted to cropland (5-year lag)	
	Agreement	At least one	Agreement	At least one
1999–2002	53	62	77	91
2003–2006	46	61	81	93
2007–2010	50	59	73	89
2011–2014	54	68	76	91
2015–2018	53	64	–	–

We conducted a cross-classification by creating composite cropland layers that combined the cropland products from the Global Land Analysis & Discovery (GLAD) group (*33*) and the European Space Agency (ESA) Climate Change Initiative (CCI) (*34*). We examined the land use in the year immediately after (2003, 2007, 2011, 2015, and 2019) deforestation occurred—as well as 5 years later—for multiple time periods (2007, 2011, 2015, and 2019) (Table 1). On the basis of cases in which at least one of the data products identified the presence of cropland, our analysis revealed that 59 to 68% of deforested areas were immediately converted to cropland and that this increased to 89 to 93% within 5 years of forest loss. As expected, in some areas, the transformation from forest to cropland was not immediate, with portions of deforested areas initially converted to pasture, grazing, or shrubland. This was visually confirmed by overlapping these areas with Google Earth images. In all, these observations confirm the strong linkages between deforestation and cropland expansion in southern Nigeria.

We then used random forest and panel fixed-effects regression models to investigate associations between annual rates of forest loss and a suite of climate anomaly variables (Table 2). We found that one-quarter (25%) of the observed variability in forest loss can be explained by climate anomalies, while the panel fixed-effects regression showed a slightly higher explanatory power (*R*^2^ = 0.31). We also ran these analyses at state level and found similar results, ranging from 15% (Benue state) to 35% (Ondo state) (Table 3), suggesting that climate variability has substantially influenced cropping decisions in certain states, particularly neighboring Ondo and Edo. These outcomes were robust across different climate datasets and spatial resolutions (tables S1, S2, and S3).

**Table 2.** Description of climate variables used in the random forest model.

Variable names	Descriptions	Unit	Source
Mean temperature	Average monthly temperature	°C	(*56*–*58*)
Maximum temperature	Average monthly maximum temperature	°C	(*56*–*58*)
Minimum temperature	Average monthly minimum temperature	°C	(*56*–*58*)
Diurnal temperature range (DTR)	The difference between the maximum and minimum temperatures over a 24-hour period.	°C	(*56*–*58*)
5-year rainfall anomaly	The average deviation of rainfall from the long-term (1986–2020) average over the previous 5 years	mm	(*54*–*57*)
10-year rainfall anomaly	The average deviation of rainfall from the long-term (1981–2020) average over the previous 10 years	mm	(*54*–*57*)

**Table 3.** Association of climate change and cropland expansion in different states. Table values show the *R*^2^ values from cross-validated random forest predictions and panel fixed effects regression at regional and state levels, using CRU climate data (*56*).

	*R*^2^ value (random forest)	*R*^2^ value (panel regression)
Whole study region	0.25	0.31
Abia	0.16	0.21
Akwa Ibom	0.16	0.2
Anambra	0.17	0.22
Bayelsa	0.18	0.23
Benue	0.1	0.15
Cross River	0.18	0.23
Delta	0.19	0.25
Ebonyi	0.17	0.22
Edo	0.28	0.34
Ekiti	0.19	0.25
Enugu	0.18	0.23
Imo	0.18	0.23
Lagos	0.21	0.27
Ogun	0.19	0.25
Ondo	0.28	0.35
Osun	0.21	0.25
Oyo	0.17	0.23
Rivers	0.18	0.23
Taraba	0.13	0.19
Kogi	0.14	0.19

**Fig. 2.**
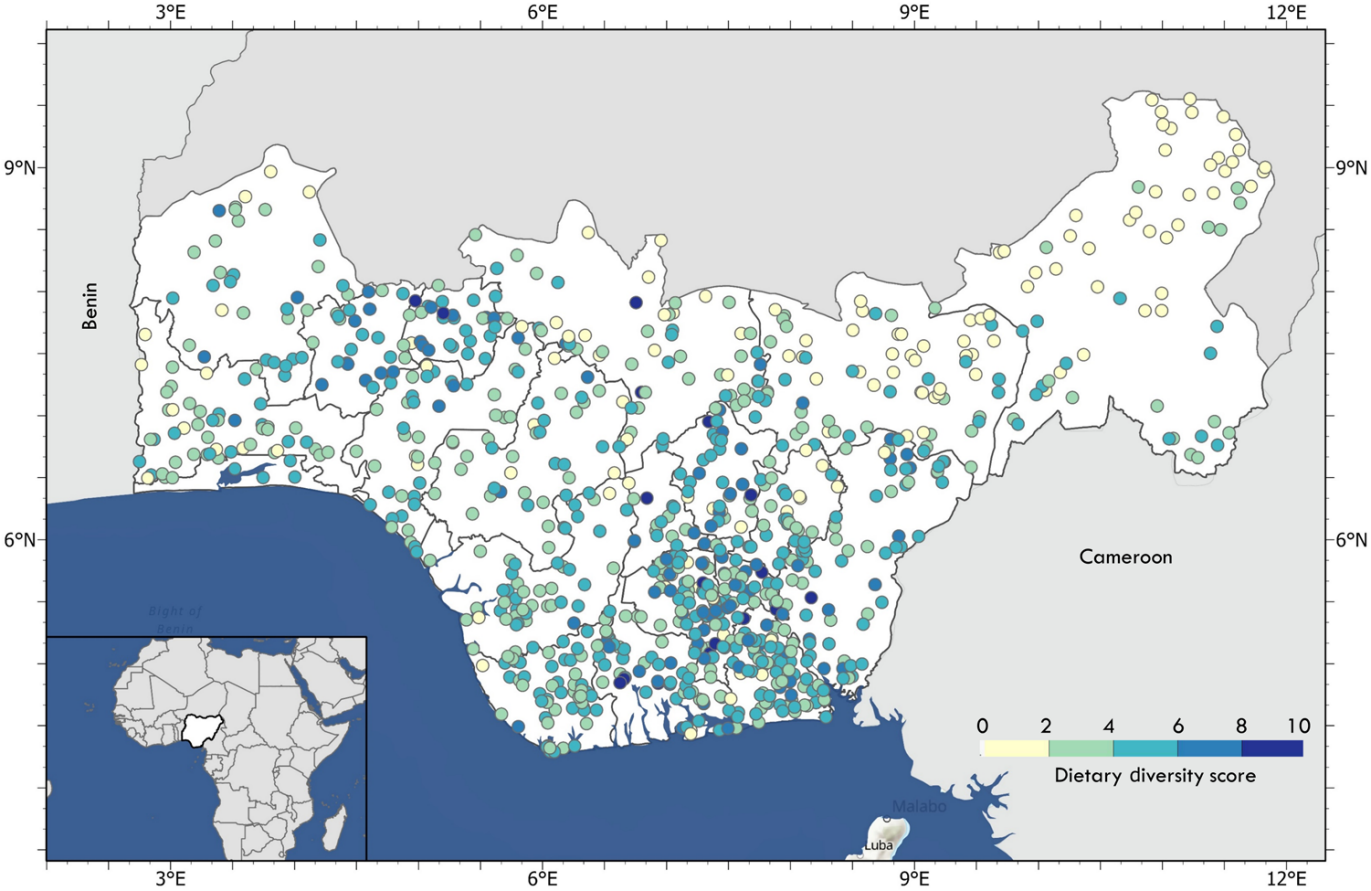
Distribution of rural cluster-level child diet diversity. Our analysis included all survey years (2008, 2013, and 2018) for which rural cluster GPS coordinates were available. Cluster color represents the average number of food groups consumed by children in the last 24 hours before the survey.

We then sought to investigate whether these forest-to-cropland conversions were associated with changes in the nutritional status of local populations. To do this, we used household-level 24-hour recall information on child dietary intakes (under 5) across 10 food groups from the Demographic and Health Survey (DHS) (*35*–*37*) to calculate georeferenced individual diet diversity scores (IDDSs) for all years (2008, 2013, and 2018) for which GPS coordinates accompanied household surveys. IDDS is a quantitative measure of food consumption that indicates household access to different types of foods and is often used as a proxy for the nutrient sufficiency of an individual’s diet (*22*, *23*, *38*, *39*). For each of the 814 georeferenced rural survey clusters (each composed of approximately 15 households), we first quantified child IDDS, finding an average score of 3.48 (3.97 in 2008, 3.03 in 2013, and 3.44 in 2018) across the study area and no clear spatial trends (Fig. 2). This falls below the minimum diet diversity score of 5 typically used to define minimum levels of adequate dietary quality (*40*), and we found that only 12% of all rural clusters met or exceeded this threshold.

For each cluster based on the DHS survey year, we then calculated initial forest cover (i.e., 10 years before the survey year), cumulative forest loss, and cumulative cropland expansion over the preceding 10-year time period. We chose a 10-year period, as 1998 represents the first reliable year of forest cover data and 2008 was the year of the first DHS survey available. We then ran linear mixed-effects regression models to examine the relationship between child IDDS and forest cover, forest loss, and cropland expansion [while controlling for a suite of covariates that are known to influence diet diversity (Table 4)].

**Table 4.** Variables used in multiple regression analysis.

Variable names	Descriptions	Source
Forest cover	The initial forest cover is the total area occupied by forests at the beginning of the study period (DHS survey year).	(*31*)
Diet diversity	FAO’s IDDS uses a scale from 0 to 10. It evaluates consumption of 10 food types: cereal grains, white roots and tubers, dark green vegetables, vitamin A–enriched vegetables and tubers, vitamin A–enriched fruits, other fruits and vegetables, meats and fish, eggs, legumes/nuts/seeds, and dairy products.	(*35*–*37*)
Time to water	Time to reach the nearest water source from a household (min)	(*35*–*37*)
Livestock density	Density of ruminant livestock within a 10-km grid cluster	(*64*)
Distance to urban area	Distance from a survey cluster to the nearest urban center	(*62*)
Distance to road	Distance from a survey cluster to the nearest road (km)	(*61*)
Population density	Average population density in a 5-km radius surrounding the cluster.	(*60*)
Child age	Age of child (months)	(*35*–*37*)
Education of household head	Education years of head of household (binary)	(*35*–*37*)
Improved toilet	Access to improved sanitation based on WHO definitions	(*35*–*37*)
Improved water	Improved water based on WHO definitions	(*35*–*37*)
Male household head	Male head of household (binary)	(*35*–*37*)
Wealth index	Composite measure of a household’s cumulative living standard, which is computed using data on a household’s ownership of selected assets (1 to 5, 5 being the wealthiest)	(*35*–*37*)
Distance to nearest hospital	Distance from a survey cluster to the nearest health care facility such as hospital or clinic (km)	(*63*)
Cropland expansion	Cumulative cropland expansion over the 10 years preceding the DHS survey	(*34*)
Cropland extent	The total area occupied by cropland at the beginning of the DHS study period	(*34*)

**Fig. 3.**
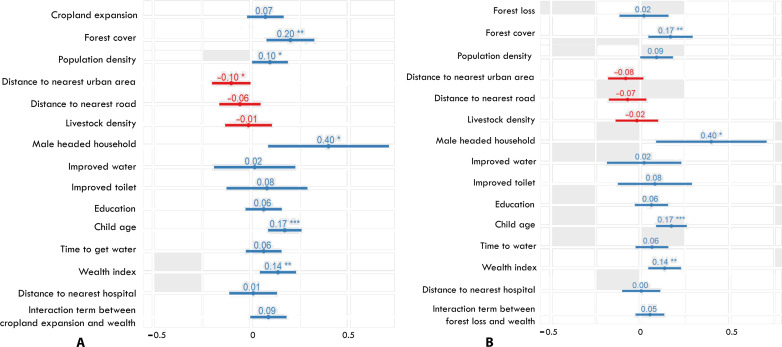
Cluster-level correlation between diet diversity and predictor variables. (A) These mixed-effects regression includes forest loss and interaction term between forest loss and wealth. (B) These mixed-effects regression includes cropland expansion and interaction term between cropland expansion and wealth. Asterisk (*) indicates statistical significance coded as ***P < 0.001, **P < 0.01, and *P < 0.05.

We found that child IDDS is positively and significantly associated with initial forest cover (Fig. 3A) and that the effect size of initial forest cover is similar in magnitude to important demographic (e.g., child age) and economic (e.g., household wealth index) determinants of child IDDS. Conversely, we found that forest loss in lieu of cropland expansion does not have a significant association with child IDDS (Fig. 3, A and B).

**Fig. 4.**
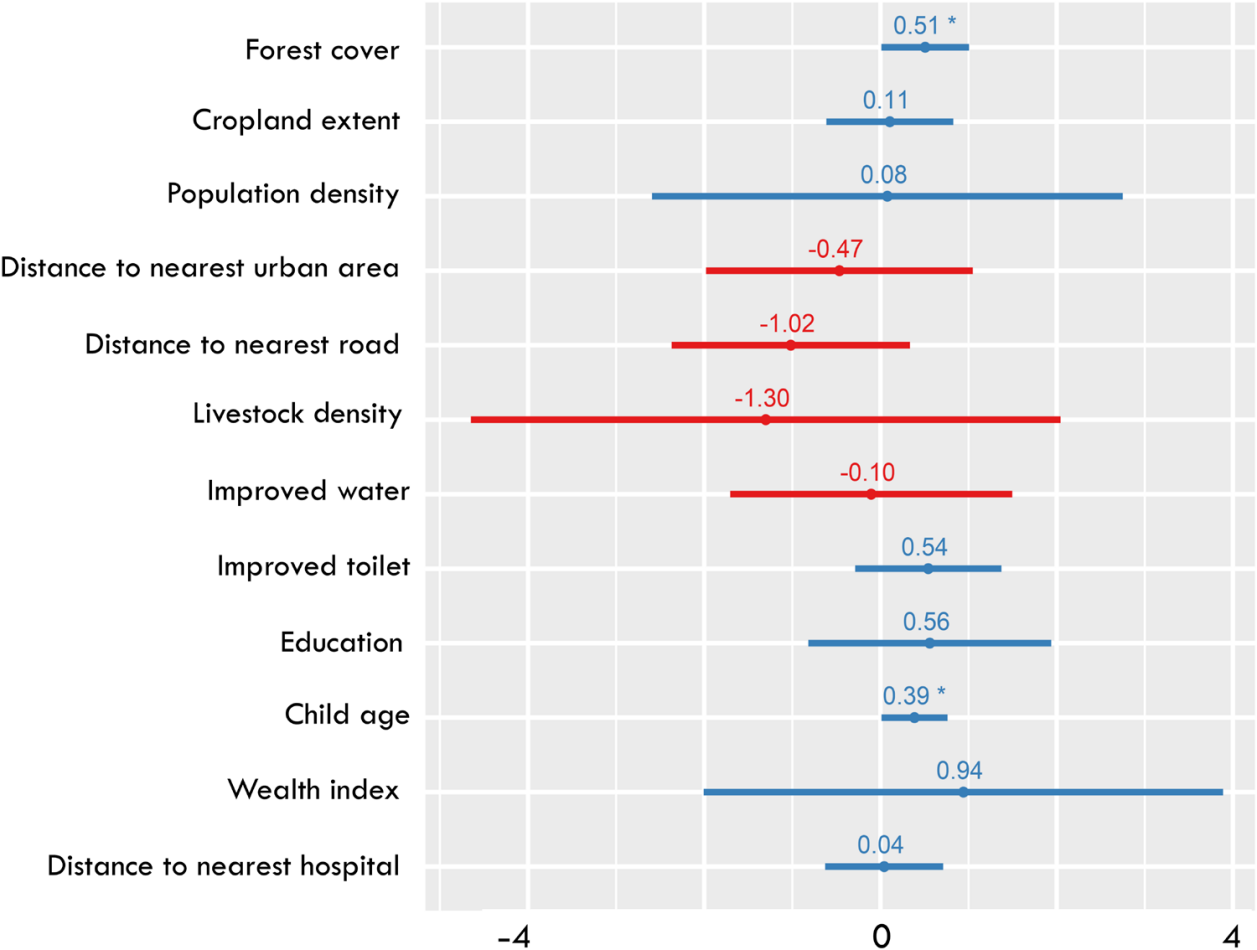
Panel fixed-effects regression examining correlation between change in forest cover, cropland extent, and diet diversity. Regression was performed at the state level. Asterisk (*) indicates statistical significance coded as ***P < 0.001, **P < 0.01, and *P < 0.05.

To better evaluate whether changes in forest cover and loss were associated with changes in diet diversity, we also performed a panel fixed-effects regression by aggregating the survey data and associated variables to the state level. After aggregating the cluster-level data to the state level, we observed a reduction in total observations to 57. Despite the lower number of observations, forest cover continued to show a positive and significant association with child IDDS. In contrast, cropland expansion and cropland extent displayed a nonsignificant relationship with IDDS, and the magnitude of their coefficients was near zero. This reaffirms that cropland expansion into deforested areas is not linked with significant improvements in nutritional outcomes (Fig. 4).

Together, these findings suggest that cropland expansion may not be an effective strategy for ensuring nutrition security in the face of climate change, as it is not providing dietary benefits that compensate for those offered by increased forest cover. As such, cropland expansion as a strategy to buffer agricultural incomes against climate variability is producing hard socio-environmental trade-offs between farmer livelihoods, rural nutrition security, and forests.

## DISCUSSION

Smallholder farmers—who account for over 90% of Nigeria’s cropland area and the vast majority of cropland expansion—face a suite of challenges in adapting to changing climate conditions (*41*). However, our analysis suggests that cropland expansion at the expense of forests—while potentially useful as a strategy to temporarily support agricultural incomes—has been insufficient for adapting diets to changing climatic conditions in smallholder settings (*42*). As such, this study challenges the conventional notion that cropland expansion automatically translates into improved nutrition security for rural communities. By combining spatially detailed information on forest change and climate variability with georeferenced household surveys, our assessment provides previously unidentified insights into the linkages between climate anomalies, cropland expansion into forests, and the dietary quality of local communities. This study highlights both a key tension that smallholder farmers face—between seeking to boost agricultural incomes while benefiting from the preservation of forests and their ecosystem services—as well as the disruptive role of climate change in deepening these economic, nutritional, and environmental trade-offs within smallholder systems. In all, our analysis suggests that cropland expansion, while offering short-term financial relief, is not associated with direct improvements in nutrition outcomes.

While most crops grown by smallholder farmers in Nigeria are consumed locally (e.g., cassava, maize, and yams), certain cash crops such as cocoa and coffee are grown primarily for export markets. This distinction highlights the dual nature of cropland expansion, where staple crops support local food security while export crops may provide income, albeit with varying effects on local nutrition. In both cases, there is an urgent need to ensure that farming activities more effectively realize benefits for the economic and nutritional well-being of rural households. Instead, our findings point to the important role of non-timber forest products (NTFPs) and other wild food resources in supporting smallholder dietary quality. Investigating whether such outcomes are consistent across diverse contexts and geographies will be an important next step of scientific inquiry.

When viewed differently, our findings point to win-win opportunities that can emerge from prioritizing nutrition-sensitive climate adaptation for farmers (*29*)—thereby enhancing and stabilizing yields, promoting more nutritious cropping choices, and protecting remaining forests. A diverse set of established and emerging strategies offer opportunities for such cobenefits for farmers and forests. Agroforestry practices can enhance soil fertility and soil moisture, reduce erosion, and allow for the simultaneous production of timber, fruits, and non–timber forest products, thereby providing farmers with multiple income streams (*43*, *44*). By leveraging the abundant freshwater resources that are physically available in southern Nigeria (*45*), expanded irrigation infrastructure can also enable farmers to boost productivity and multicropping on existing croplands (*46*), buffer yields against rising climate variability, and better predict harvests (*47*). Enhancing the physical and economic access of farmers to fertilizers and improved seeds (with a focus on drought- and pest-tolerant varieties) is another potentially transformative (but underused) strategy to amplify crop production, avoid cropland expansion, and reduce climate impacts (*48*, *49*). Regarding these potential interventions, the federal government has recently enacted several policies aimed at enhancing agricultural productivity. Among these initiatives was the Agricultural Transformation Agenda, which promoted the use of high-yielding crop varieties, thereby incentivizing farmers to adopt improved seeds (*50*, *51*). In addition, the Green Alternative program, spanning from 2016 to 2020, was introduced to foster sustainable agricultural practices and the incorporation of modern farming technologies (*52*). The Presidential Farmers Initiative, launched in 2016, provided farmers with complimentary fertilizer to support their cultivation efforts (*52*). In addition, the Anchor Borrowers Program, established by the Nigerian Central Bank, offered loans to smallholder farmers to assist in acquiring agricultural inputs (*53*). Yet, the benefits of these programs have often not materialized as intended, and the programs themselves have often been short-lived.

While there continues to exist great scope for achieving socioeconomic and environmental win-wins through these interventions, the widespread adoption of all these strategies will require substantial and sustained funding and coordination on the part of government and development agencies. Without dedicated financial support coupled with sustainable efforts at capacity strengthening and knowledge transfer, farmers cannot on their own be expected to enact wholesale change in their efforts to adapt to variable climate conditions. Given the rapid population growth (and associated rise in food demand) expected in Nigeria in the coming decades, well-designed investments in agricultural intensification can not only help to ensure future food security and nutrition but also promote widespread rural development and protect the country’s remaining forests and other natural spaces. This ability to realistically incorporate multiple dimensions into intervention planning and implementation is the crux of sustainable food production systems and—more broadly—can contribute substantially toward achieving multiple sustainable development targets in tandem.

## MATERIALS AND METHODS

Our study focuses on the 20 southern states (Abia, Akwa Ibom, Anambra, Bayelsa, Benue, Cross River, Delta, Ebonyi, Edo, Ekiti, Enugu, Imo, Kogi, Lagos, Ogun, Ondo, Osun, Oyo, Rivers, and Taraba) (Fig. 1) of Nigeria, where 98.9% of the country’s remaining forest cover occurs. The region is characterized by a variety of ecological zones including mangrove forests, rainforests, and savannah woodlands. There is a distinct wet season (beginning in March and extend until October) with annual rainfall ranging between 1500 and 4000 mm across the study area. The region typically has a warm climate year-round, with temperatures ranging between 25° and 30°C. Agro-ecological conditions in the region are conducive to the cultivation of crops such as yams, cassava, maize, rice, cocoa, oil palm, rubber, and various fruits and vegetables.

### Data

#### Climate variables

Precipitation datasets included in this study were Climate Hazards Group InfraRed Precipitation with Station (CHIRPS v2.0) (spatial resolution: 0.05°; temporal resolution: daily; time period: 1981 to 2021) (*54*), Tropical Rainfall Measuring Mission (spatial resolution: 0.25°; temporal resolution: daily; time period used: 1998 to 2015) (*55*), Climate Research Unit (CRU) v3.23 (spatial resolution: 0.5°; temporal resolution: daily; time period used: 1981 to 2021) (*56*), and Willmot-Matsura (WM) (spatial resolution: 0.5°; temporal resolution: monthly; time period used: 1991 to 2017) (*57*). Temperature datasets used in this study were Berkeley Earth Surface Temperature (spatial resolution: 1°; temporal resolution: monthly; time period used: 1989 to 2021) (*58*), CRU v3.23 (spatial resolution: 0.5°; temporal resolution: daily; time period used/available: 1989 to 2021) (*56*), and WM (spatial resolution: 0.5°; temporal resolution: monthly; time period used/available: 1989 to 2017) (*57*). These datasets were used to calculate a suite of variables to quantify climate anomalies (Table 1). The mean, minimum, and maximum daily temperatures were determined by calculating temporal averages of the monthly temperature, monthly minimum temperature, and monthly maximum temperature, respectively, over the course of the main growing season (March to October) (*59*). Diurnal temperature range was calculated as the difference between the maximum and the minimum temperatures recorded over a 24-hour period. Five-year and 10-year rainfall anomalies were calculated as the mean deviation from the long-term average rainfall over the prior 5 and 10 years, respectively.

#### Demographic and health surveys

Much of the data used in this study came from the DHSs (*35*–*37*), which are representative household-level survey data on demography, health, and nutrition. We used all DHS survey years (2008, 2013, and 2018) for which georeferenced data were available. The DHS variables of interest were child age, wealth index, time to water, education of household head, improved toilet, improved water, and 24-hour recall for 10 food groups (which asks the respondents to recall what they feed their children under five in the previous 24 hours). This 24-recall variable is a qualitative measure of food consumption that indicates household access to different types of foods and is also a proxy for nutrient sufficiency of the diet of individuals (*39*). We used this information to calculate our outcome variable of interest—IDDS for each child under 5. IDDS is calculated based on the United Nations Food and Agriculture Organization (UN FAO) (*38*), and is a scale ranging from 1 to 10 based on the intake of 10 different types of food groups including (i) cereal grains, (ii) white tubers and root foods, (iii) dark leafy greens, (iv) vitamin A–rich vegetables/tubers, (v) vitamin A–rich fruits, (vi) other fruits and vegetables, (vii) meat and fish foods, (viii) eggs, (ix) legumes/nuts/seeds, and (x) milk and milk products. Other variables used in the study are described in the section immediately below.

#### Covariates of diet diversity

We obtained population density data from the NASA Socioeconomic Data and Applications Center (SEDAC) (*60*) database at a spatial resolution of 30 arc sec (1 km). The year of the data used for an individual cluster depended on the year of the DHS survey—2005 if the survey year was 2008, 2010 if the survey year was 2013, and 2015 if the survey year was 2018. We averaged the population density over a 5-km radius for each DHS cluster. We calculated distance to roads using the Global Distribution of Roads dataset from NASA SEDAC (*61*) around the year 2000. We calculated distance to urban centers using the Global Urban Extent (v1) from NASA SEDAC (*62*) for the year 1995. We calculated distance to the nearest health care facility using the Humanitarian OpenStreetMap health care facilities dataset, which provided the geographic location of hospitals, clinics, and other primary care provider facilities. These data were used to measure the proximity of each DHS cluster to the nearest health care facility such as hospital and clinic (*63*). We calculated livestock density data using the Gridded Livestock of the World (GLW3) database (*64*), which provides the global population densities of cattle, buffaloes, horses, sheep, goats, pigs, chickens, and ducks for the year 2010. In each country and for each type of livestock, a livestock unit was calculated by taking the ratio of production in tons to the number of animals produced/slaughtered (*20*). This factor was then multiplied by the population density rasters of each animal, and all rasters were added to obtain the total livestock unit for each grid cell. We averaged the livestock density within 10 km of each DHS cluster and categorized it into eight different quantile-based classes based on the distribution of the data for each country.

#### Forest cover and loss

Gridded (30 m) data on forest cover and annual forest loss between the years 1998 and 2021 were obtained from the Tropical Moist Forest (TMF) dataset (*31*). Initial forest cover was defined as any 30 m by 30 m pixel having a tree cover percentage of greater than 50% in the year 1998. All locations of known tree plantations (i.e., not native forests) were excluded from our analysis. A pixel was defined as experiencing forest loss when the pixel category changed from undisturbed in year *t* to degraded in year *t* + 1. We assumed that any pixel experiencing forest loss underwent complete forest loss and remained unforested for the remainder of the study period. We did not consider forest gain in our calculations of deforestation rates as areas of forest gain in the study region are much smaller than those of forest loss (3.1 Mha). We assume that all forest loss occurred as a result of cropland expansion—an assumption supported by the literature (*16*, *18*) and confirmed by cross-classification (table S4) of TMF data with global cropland data (30 m) (*65*).

#### Cropland extent and cross-classification between forest loss and cropland expansion

We used the GLAD cropland data product (*33*) and the ESA CCI cropland dataset, which spans 1992 to 2020 at a 300-m spatial resolution (*34*). The GLAD dataset is a gridded cropland dataset with a 30-m spatial resolution, representing a globally consistent cropland extent time series (*33*). The dataset spans 2000 to 2019, with cropland mapping performed using Landsat satellite data, transformed into multitemporal metrics for machine learning classification. The mapping was conducted in 4-year intervals (2000 to 2003, 2004 to 2007, 2008 to 2011, 2012 to 2015, and 2016 to 2019), resulting in five cropland layers (2003, 2007, 2011, 2015, and 2019). For cross-classification between forest loss and cropland expansion, we combined the GLAD and ESA CCI cropland datasets into composite cropland map corresponding to the years (2003, 2007, 2011, 2015, and 2019), in accordance with the time windows of the GLAD dataset. Cross-classification was performed for deforested areas immediately converted to cropland in the subsequent year and deforested areas converted to cropland within 5 years of forest loss [to account for more indirect pathways (e.g., forest to pasture to cropland)]. Given the well-known limitations of global cropland products in accurately classifying smallholder croplands (*66*, *67*) and the fact that each cropland product is trained using a different set of crops as groundtruth, we also assessed the fraction of forest-to-cropland conversion that occurred where both cropland products spatially agreed (i.e., “Agreement”) or where at least one of the products indicated the presence of cropland (i.e., “At least one”). We did not assess the 2015 to 2018 deforested area for the 5-year time lag analysis as there was no corresponding cropland loss data from 5 years later of 2023. This approach allowed us to quantitatively assess the extent of deforested areas converted into cropland over time.

### Modeling associations between forest loss, cropland expansion, and climate anomalies

We applied a random forest machine learning algorithm (*68*) to evaluate the correlation between annual rates of forest loss and a suite of climate variables (Table 1). Random forest is a nonparametric statistical method that uses decision trees to perform regressions or classifications and is robust to overfitting (*68*, *69*). This method has been previously applied in the analysis of agricultural outcomes (including planted area) in association with climate variables (*4*, *70*, *71*). All data were randomly partitioned into an 80-20% split for training and validation (*42*, *72*). Hyperparameters (i.e., the number of trees to build, maximum depth of the tree, minimum leaf node size, and number of features to use for splitting) used in each model were tuned based on a grid search approach (*73*). To estimate and compare the forest loss variance explained by climate variables, we calculated *R*^2^ values from cross-validated predictions. This analysis was repeated for the entire study region as well as for each state (Table 3).

To improve the explanatory power of the model, we also conducted a panel fixed-effects regression incorporating both spatial (grid-level) and temporal (yearly) fixed effects (Eq. 1). This approach allowed us to control for unobserved heterogeneity across regions and time-invariant characteristics. In addition, we included baseline forest cover as a fixed effect to account for the initial forest conditions in each grid

$$\begin{array}{c} \text{Forest loss}=\beta +\beta_{0}\;\left(5-\text{year rainfall anomaly}\right)\\ +\beta_{1}\left(10-\text{year rainfall anomaly}\right)\\ +\beta_{2}\left(\text{mean temperature}\right)\\ +\beta_{3}\left(\text{maximum temperature}\right)+\beta_{4}\left(\text{minimum temperature}\right)\\ +\beta_{5}\left(\text{diurnal temperature range}\right)\\ +\beta_{6}\left(\text{diurnal temperature range}\right)\\ +b_{1j}\left(\text{grid and year fixed effects}\right)+\in \end{array}$$ (1)

where β is the intercept, β_0_, β_1_, …, β_6_ coefficients represent the fixed effects of each independent variable, *b*_1*j*_ represents the state and year fixed effects, and ∈ is the error term.

### Regression modeling of associations between forest loss, cropland expansion, and child diet diversity

Using multilinear mixed-effects regression models (Eq. 1) (*74*, *75*), we assessed the relationship between forest loss (in lieu of cropland expansion) and IDDS, while controlling for multiple agroecological, geographic, and socioeconomic variables that are known to influence diet diversity (Eq. 2). We used the survey year as a random effect in our model. In total, our model contained data from 814 DHS clusters (*N*)

$$\begin{array}{c} \text{Individual diet diversity score}\;\left(\text{IDDS}\right)=\beta \\ +\beta_{0}\;\left(\text{cumulative forest loss in the preceding}\;10\;\text{years}\right)\\ +\beta_{1}\left(\text{forest cover}\right)\\ +\beta_{2}\;\left(\text{distance to urban center}\right)+\beta_{3}\;\left(\text{distance to road}\right)\\ +\beta_{4}\;\left(\text{population density}\right)\\ +\beta_{5}\;\left(\text{livestock index}\right)+\beta_{6}\;\left(\text{age}\right)\\ +\beta_{7}\;\left(\text{education of household head}\right)\\ +\beta_{8}\;\left(\text{improved toilet}\right)+\beta_{9}\;\left(\text{improved water}\right)\\ +\beta_{10}\;\left(\text{sex}\;\text{of the household head}\right)\\ +\beta_{11}\;\left(\text{time to water source}\right)+\beta_{12}\;\left(\text{wealth index}\right)\\ ++\beta_{13}\;\left(\text{distance to nearest hospital}\right)\\ +\beta_{14}\;\left(\text{interaction term between forest loss and wealth}\right)\\ +b_{1j}\left(\text{Year}\right)+\in \end{array}$$ (2)

where β is the intercept, β_0_, β_1_, …, β_13_ coefficients represent the fixed effects of each independent variable, *b*_1*j*_ represents the random effects for different years, β_14_ represents the regression coefficient for the interaction term between forest loss and forest cover, and ∈ is the error term. This cluster-level mixed-effects model was also repeated by replacing cumulative forest loss with cumulative cropland expansion.

In addition, to capture temporal changes and control for unobserved heterogeneity over time and space, we aggregated the cluster-level data to the state level and structured the dataset as a panel. We then conducted a panel fixed-effects regression to analyze the direct relationship between change in forest cover, change in cropland extent, and change in nutritional outcomes (IDDS), while accounting for both state and time fixed effects (Eq. 3). This approach allowed us to control for time-invariant factors at the state level as well as annual variations, providing a more rigorous assessment of the association of forest loss and cropland expansion with diet diversity change

$$\begin{array}{c} \text{Individual Diet Diversity Score}\;\left(\text{IDDS}\right)\\ =\beta +\beta_{0}\;\left(\text{cropland extent}\right)\\ +\beta_{1}\;\left(\text{forest cover}\right)+\beta_{2}\;\left(\text{distance to urban center}\right)\\ +\beta_{3}\;\left(\text{distance to road}\right)\\ +\beta_{4}\;\left(\text{population density}\right)+\beta_{5}\;\left(\text{livestock index}\right)+\beta_{6}\;\left(\text{age}\right)\\ +\beta_{7}\;\left(\text{education of household head}\right)+\beta_{8}\;\left(\text{improved toilet}\right)\\ +\beta_{9}\left(\text{improved water}\right)++\beta_{10}\;\left(\text{wealth index}\right)\\ +\beta_{11}\;\left(\text{distance to nearest hospital}\right)\\ +b_{1j}\;\left(\text{state and year fixed effects}\right)+\in \end{array}$$ (3)

where β is the intercept, β_0_, β_1_, …, β_13_ coefficients represent the effects of each independent variable, *b*_1*j*_ represents the state and year fixed effects, and ∈ is the error term.
