# Supplementary material for: Cropland expansion links climate extremes and diets in Nigeria
Source: Sci Adv. 2025 Jan 10;11(2):eado5541. doi: 10.1126/sciadv.ado5541 (PMC11721561; doi:10.1126/sciadv.ado5541)
Supplement: Supplementary file 1 — Tables S1 to S3 [file sciadv.ado5541_sm.pdf]

Science Advances
AAAS

Supplementary Materials for

Cropland expansion links climate extremes and diets in Nigeria

Bhoktear Khan *et al.*

Corresponding author: Bhoktear Khan, bhoktear@udel.edu

Sci. Adv. **11**, eado5541 (2025)
DOI: 10.1126/sciadv.ado5541

This PDF file includes:

Tables S1 to S3

Table S1. Association between climate anomalies and cropland expansion using a 10-km grid size for aggregation. Table values show the R^2 values from cross-validated random forest predictions.

		Precipitation dataset			
		CHIRPS	CRU	TRMM	WM
**Temperature dataset**	BEST	0.20	0.17	0.12	0.17
	CRU	0.18	0.25	0.11	0.17
	WM	0.18	0.13	0.11	0.16

Table S2. Association between climate anomalies and cropland expansion using a 25-km grid size for aggregation. Table values show the R^2 values from cross-validated random forest predictions.

		**Precipitation dataset**			
		CHIRPS	CRU	TRMM	WM
**Temperature dataset**	BEST	0.15	0.17	0.12	0.12
	CRU	0.17	0.18	0.11	0.13
	WM	0.13	0.11	0.09	0.12

Table S3. Association between climate anomalies and cropland expansion using a 50-km grid size for aggregation. Table values show the R^2 values from cross-validated random forest predictions.

		**Precipitation dataset**			
		CHIRPS	CRU	TRMM	WM
**Temperature dataset**	BEST	0.11	0.13	0.11	0.13
	CRU	0.12	0.16	0.09	0.15
	WM	0.08	0.16	0.09	0.11
